# The role of biochar in combating microplastic pollution: a bibliometric analysis in environmental contexts

**DOI:** 10.3762/bjnano.16.102

**Published:** 2025-08-21

**Authors:** Tuan Minh Truong Dang, Thao Thu Thi Huynh, Guo-Ping Chang-Chien, Ha Manh Bui

**Affiliations:** 1 Department of Environmental and Safety Engineering, Dayeh University, Changhua County 510006, Taiwan; 2 Institute of Environmental Toxin and Emerging-Contaminant Research, Cheng Shiu University, Kaohsiung 833301, Taiwan; 3 Super Micro Mass Research and Technology Center, Cheng Shiu University, Kaohsiung 833301, Taiwan; 4 Center for Environmental Toxin and Emerging-Contaminant Research, Cheng Shiu University, Kaohsiung 833301, Taiwan; 5 Faculty of Engineering and Technology, Saigon University, 273 An Duong Vuong Street, Cho Quan Ward, Ho Chi Minh City 700000, Vietnam

**Keywords:** aquatic pollution, bibliometric analysis, biochar, microplastics (MPs), soil remediation

## Abstract

This study employs a bibliometric analysis using CiteSpace to explore research trends on the impact of biochar on microplastics (MPs) in soil and water environments. In agricultural soils, MPs reduce crop yield, alter soil properties, and disrupt microbial diversity and nutrient cycling. Biochar, a stable and eco-friendly material, has demonstrated effectiveness in mitigating these effects by restoring soil chemistry, enhancing microbial diversity and improving crop productivity. Recent studies report that biochar increases crop yields by 30–81%, even under high MP contamination levels (up to five times that of biochar-modified bacteria). Additionally, biochar enhances Olsen-P availability by 46.6%, increases soil organic carbon in microaggregates by 35.7%, and reduces antibiotic resistance genes by promoting beneficial microbes such as *Subgroup 10*, *Bacillus*, and *Pseudomonas*. In aquatic systems, biochar serves as an efficient adsorbent, particularly for MPs larger than 10 µm, including polystyrene. Studies suggest that fixed-column models achieve superior removal efficiency (95.31% ± 5.26%) compared to batch systems (93.36% ± 4.92%). Specifically, for MPs ≥10 µm, fixed columns reach 99% efficiency, while magnetically modified biochar captures 96.2% of MPs as small as 1 µm. These efficiencies stem from biochar’s integration of physical and chemical mechanisms that enhance MP retention, particularly for MPs smaller than 10 µm, positioning it as a promising solution for nanoplastic remediation.

## Introduction

Plastics are widely utilized in various industries and daily life due to their low production cost. The average global per capita consumption of plastic is 60 kg/year, with Europe exhibiting a significantly higher rate of 150 kg/year [1]. However, the rapid increase in plastic consumption is accompanied by the generation of substantial plastic waste. For example, China, the leading producer and consumer of plastic, generated 26.74 million tons of plastic waste in 2019, with projections indicating an increase to 34.82 million tons by 2035 [2]. Within Europe, Ireland has the highest per capita plastic packaging waste generation, approximately 67 kg/year [3]. Despite efforts to recycle plastic, only 9% of virgin plastic was recycled in 2015, with 12% incinerated and 79% accumulating in landfills or the natural environment [4].

Microplastics (MPs) have garnered significant attention due to their adverse impacts on human health and the ecological environment. Research primarily focuses on identifying their presence, risks, and sources in the environment and biota. Although MPs are produced in large quantities, effective removal techniques remain underdeveloped [5–6]. Recent studies have confirmed the widespread presence of MPs in agricultural soils and water bodies, highlighting their environmental and human health risks. In Yan’an, China, MP concentrations in agricultural soils reached 4505 ± 435 ng/kg, with polyethylene (PE) accounting for 37.4% of the total [7]. Similarly, in the United Kingdom, plastic-mulched soils contained 3680 ± 129.1 particles/kg, while non-mulched soils exhibited lower levels at 2667 ± 84.1 particles/kg [8]. The primary sources of MPs in agricultural soils include irrigation, fertilizers, farming practices (e.g., plastic mulching), and atmospheric deposition [5–6]. Additionally, recreational soils have been reported to contain higher MP concentrations than agricultural soils, as observed in Indore City, India [9].

MP contamination extends to water bodies, where seasonal variations influence the distribution of MPs. In South African rivers such as Crocodile and Luvuvhu, MP concentrations in water were higher during the dry season (5.4 particles/L) than in the wet season (3.3–4.3 particles/L), while sediment contamination was more severe during the wet season, with contamination factors categorized as “very high” (15.6) in wet periods and “high” (4.9) in dry periods [10]. Similarly, effluent from wastewater treatment plants contained 192 particles/L for 2 µm MPs and 323 particles/L for 10 µm MPs, underscoring the role of treatment facilities as pathways for MP release into aquatic environments [11].

The ecological and human health risks associated with MPs necessitate urgent mitigation strategies. In Yan’an, China, MP pollution loading indices ranged from 1.00 to 2.48, indicating light ecological pollution in agricultural soils [7]. In contrast, wastewater systems in Oman exhibited polymer indices ranging from moderate to extreme danger, emphasizing the need for enhanced MP removal technologies [12]. These findings highlight the pervasive nature of MP contamination across terrestrial and aquatic systems, reinforcing the importance of targeted remediation approaches.

To address MP contamination, various adsorbent materials have been investigated. Granular activated carbon at a concentration of 1.5 g/L has demonstrated adsorption efficiencies of up to 90% for MP fragments and fibers. Additionally, three-dimensional graphene oxide has shown adsorption capacities of up to 617.28 mg·g^−1^ for polystyrene MPs of 5 µm in size [13–14]. The integration of adsorbents with appropriate treatment models has further enhanced removal efficiency. For instance, coal gasification slag-based adsorbents combined with fluidized bed treatment achieved 99.2% MP removal, while granular activated carbon coupled with a fixed-column system attained 95.2% removal efficiency [15–16].

Biochar (BC) has emerged as a promising material for environmental remediation, offering benefits such as pollutant adsorption, soil improvement and climate mitigation. Life cycle assessments indicate that BC application can result in climate benefits ranging from −1.4 to −0.11 tonnes CO_2_-eq per tonne of biochar [17]. Its versatility extends to removing pollutants, enhancing plant growth, and decolorizing organic dyes in wastewater [18–21]. Recent studies exploring the use of BC for MP remediation have yielded promising results, further emphasizing its potential for addressing environmental contamination.

Recent evaluations of MP removal methods have classified biological, physicochemical, and biochar-based techniques according to their treatment models, such as batch and fixed-column systems [22]. Modified biochars have been developed to enhance MP capture efficiency, including magnetic biochar, which facilitates easy separation from aqueous environments [22–23]. A comparative study by Mulindwa, et al. [24] assessed different biosorbents, including biochar, sponge/aerogel biomass-derived materials, and biomass-based graphene materials, revealing that biochar exhibits comparable efficiency to sponge/aerogel biomass-derived materials. Furthermore, while MP-induced soil alterations have shown positive responses in terms of enzyme activity [22,25], existing studies did not comprehensively assess MP removal efficiencies across different size ranges in aqueous environments, nor have they extensively evaluated the broader impact of MP contamination on soil ecosystems using BC. The role of modified BC functional groups in MP remediation also remains insufficiently explored. Moreover, bibliometric approaches have not been widely applied to visualize research trends in BC applications for MP mitigation.

To advance the understanding of MP pollution and biochar’s remediation mechanisms, bibliometric analysis using CiteSpace has proven instrumental. CiteSpace facilitates citation network visualization, co-citation analysis and the identification of emerging research trends, providing insights into the intellectual structure of this scientific domain [26]. By uncovering key contributors and trends, bibliometric tools support informed policy making and research prioritization. The application of bibliometric analysis enables an accessible and comprehensive visualization of research dynamics and development trends in BC-based MP remediation. Additionally, using authoritative journal databases such as “Social Sciences Citation Index” (SSCI), “Social Science Information” (SCI), and “Arts and Humanities Citation Index” (AHCI) ensures objectivity and scientific rigor, minimizing subjective biases and providing a broad perspective on research directions.

Thus, this study aims to (i) identify key research areas by analyzing topics, keywords, and relevant studies on “MPs and BC” through bibliometric methods, (ii) synthesize existing literature on major research themes, including modified biochar synthesis and its role in remediating contaminated environmental matrices, and (iii) expand the assessment of BC and modified BC in mitigating MP-contaminated soil by evaluating crop yield, microbial expression, gene activity, and enzymatic responses, while also analyzing the effectiveness of various modified biochars in removing MPs of different sizes in aqueous environments. The findings of this review provide critical insights into the current state and future directions of biochar's application in addressing MP pollution.

## Review

### Research methodology and analytical framework

#### Data collection and processing

The study employed bibliometric analysis using CiteSpace to examine publication trends and research topics related to MP removal with BC. CiteSpace was chosen for its ability to detect citation bursts and perform cluster labeling analysis, highlighting emerging research trends and intellectual structures. Compared to VOSviewer, which primarily visualizes co-authorship networks and keyword co-occurrences, CiteSpace provides advanced temporal and structural metrics, such as betweenness centrality, to map pivotal connections and identify transformative shifts in scientific literature. While VOSviewer excels in intuitive knowledge mapping over time, CiteSpace offers deeper insights into the dynamic evolution of research fields [27].

**Figure 1:**
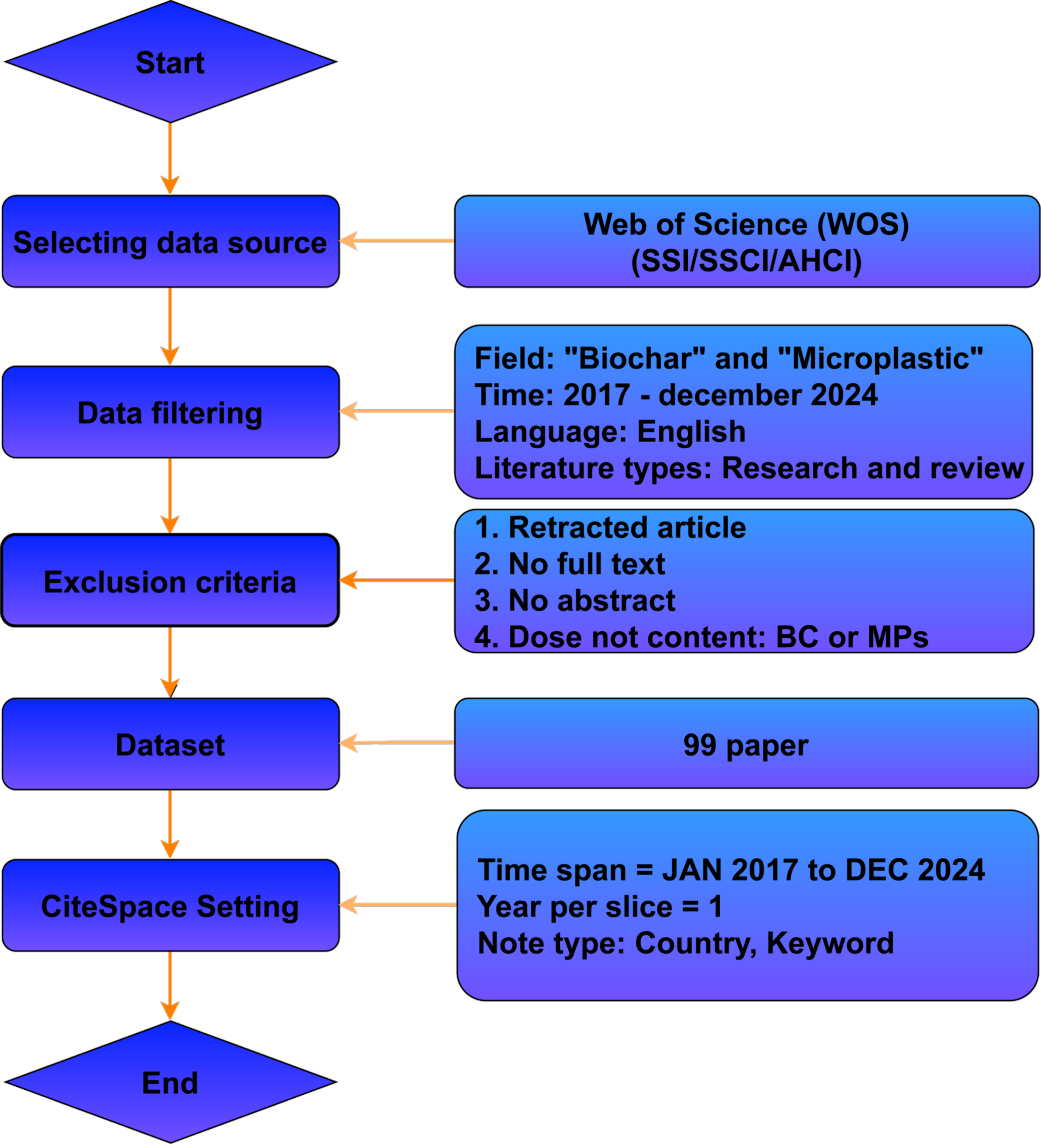
Data processing workflow and methodological processes.

Bibliometric data were collected from the “Web of Science” (WOS) database to ensure comprehensive coverage and avoid the omission of relevant articles indexed in only one source. The data collection spanned January 2017 to December 2024, with the search process initiated at 02:59. Relevant keywords such as “microplastic” and “biochar”, along with their abbreviations, were used to capture a broad spectrum of literature. The dataset comprised 99 peer-reviewed journal articles, excluding conference papers, reviews, book chapters, editorials, errata, and comments to maintain credibility and research quality. Articles published before 2017 were excluded to focus on recent advancements and eliminate outdated or unreported studies. The procedure is illustrated in Figure 1.

#### Bibliometric analysis

The bibliometric analysis utilized citation and co-citation analysis to visualize knowledge structures and research trends. Quantitative techniques, including descriptive statistics, citation analysis, keyword clustering, and trend analysis, were applied to assess research impact and institutional collaborations. Higher citation frequencies indicated greater influence and stronger collaborative patterns. The trend analysis identified thematic clusters, research hotspots, and emerging frontiers, offering a comprehensive understanding of the field’s development.

In the knowledge mapping process, the node size represents the frequency of references or citations, while edge thickness reflects the strength of relationships between nodes [28]. The betweenness centrality of a node was defined using the Kleinberg burst detection algorithm, measuring the node’s influence within the network. The centrality value δs*ν was calculated as:


[1] $$\delta_{s*}\left(v\right)=\sum_{t\in v}\delta_{st}\left(v\right)=\sum_{w:v\in P_{x}\left(w\right)}\frac{\sigma_{sv}}{\sigma_{sw}}\left(1+\delta_{s*}\left(w\right)\right).$$

Nodes with high centrality values are indicative of key research topics and critical publications, distinguishing them as influential elements within the scientific domain. The intensity of hotspots is reflected in node frequency, while centrality values quantify a node’s relative importance [29].

#### Data filtering and quality control

To preserve analytical validity, articles irrelevant to the study’s scope were manually excluded based on their titles and abstracts. Exclusion criteria included retracted articles, those without full text or abstracts and studies lacking relevance to biochar (BC) or microplastics (MPs). The selected articles were systematically reviewed, categorized, and analyzed using bibliometric software. Data visualization facilitated the identification of research trends and relationships among publications, contributing to a clearer understanding of the field’s progression.

### Current status and thematic analysis

#### Bibliometric analysis

**Figure 2:**
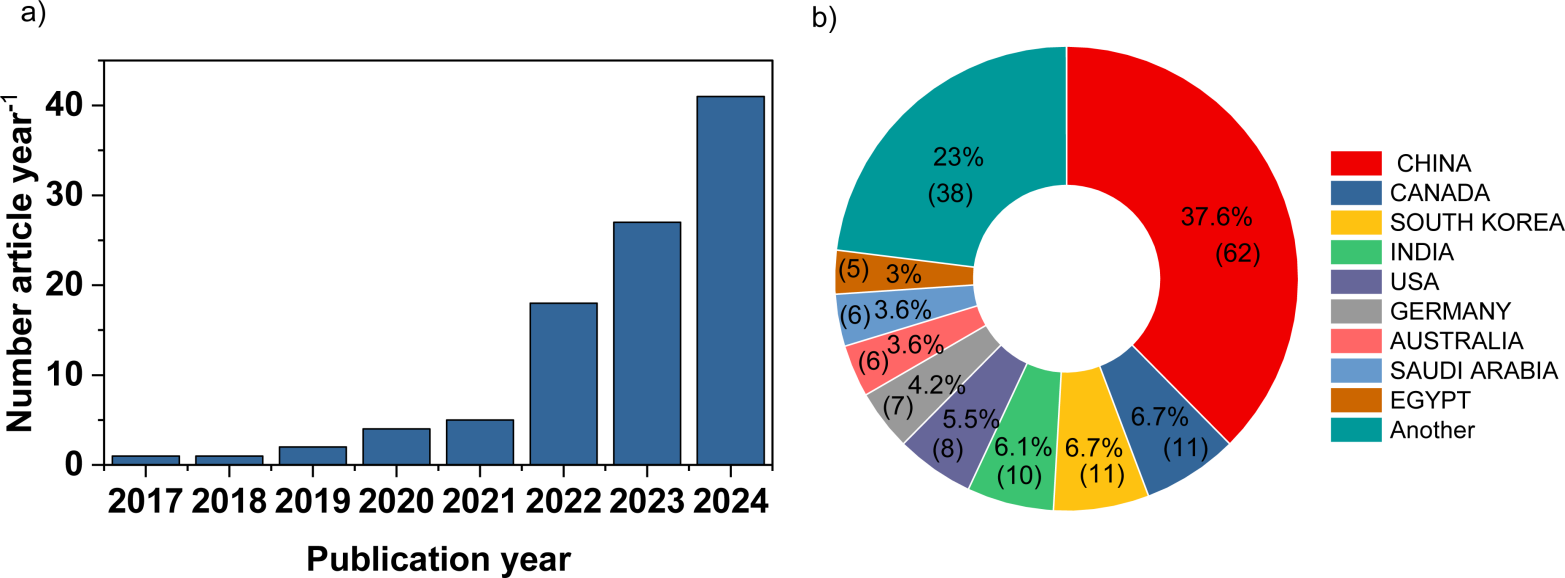
(a) Number of publications on BC and MPs per year and (b) top ten contributing nations worldwide.

**Publication trends:** The annual number of publications and the geographical distribution of the authors publishing research on BC and MPs across the globe are illustrated in Figure 2. Research in this field began with one publication per year in 2017 and 2018, increasing to two publications in 2019, followed by four and five publications per year in 2020 and 2021, respectively. By 2024, the number of publications had surged to 41, a tenfold increase compared to 2020. This sharp growth underscores the rising interest of researchers and the scientific community in BC and MP as pressing and engaging research topics.

China is the leading contributor with 62 publications, followed by Canada, South Korea, and India. The significant research output from Asian countries highlights a growing regional focus on addressing the environmental and scientific challenges related to BC and microplastics MPs. As shown in Figure S1, Supporting Information File 1, four primary research themes have been explored at the national level since 2017, namely, (i) adsorption capacity and mechanisms, (ii) composting-related fertilizer applications, (iii) degradation of plastic mulch into polymers in soil, and (iv) pathways of plastic decomposition.

**Figure 3:**
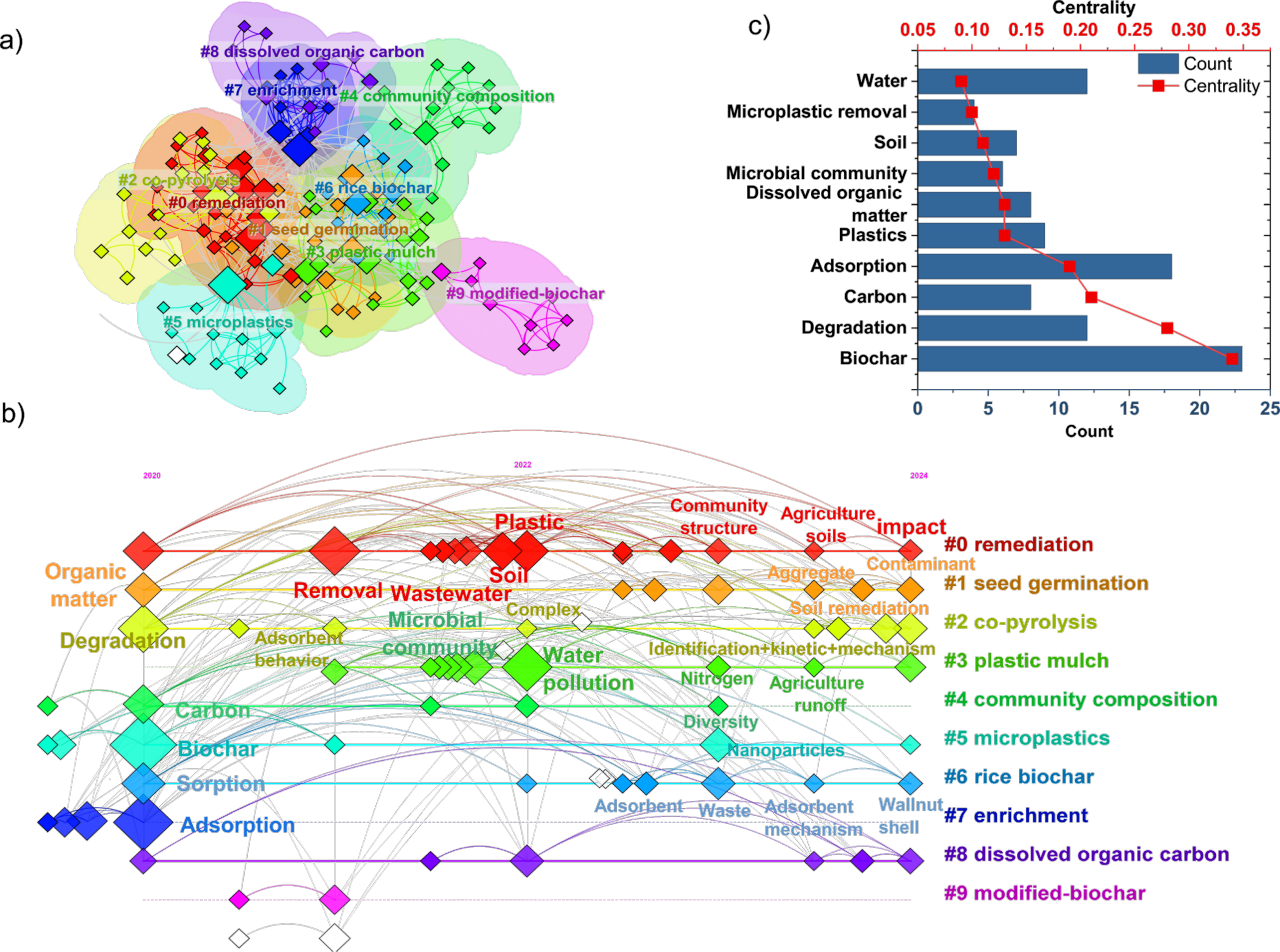
(a) Bibliometric network visualization of the published articles, (b) co-occurrence clustering map of keywords, and (c) chart illustrating centrality and count indices across 154 items.

**Trends and hotspot analysis:** Using an AI-based bibliometric tool, a total of 99 publications were analyzed, resulting in the identification of 154 items grouped into nine primary keywords, that is, remediation, seed germination, co-pyrolysis, plastic mulch, community composition, MPs, rice biochar, enrichment, dissolved organic carbon, and modified biochar. As depicted in Figure 3a, the clustering and proximity of these items indicate frequent co-citation among the publications. Their temporal evolution is visualized in Figure 3b, while Figure 3c shows the frequency and connectivity of each keyword within the network. Among the top ten items with the highest centrality, “biochar” emerges as the core linking element across various research domains related to mitigating MP impact. The second most central item, “degradation”, has been a focal point since 2020, primarily examining the influence of MPs on soil organic matter and the role of biodegradable plastics, supported by biochar, in MP breakdown. Items such as “adsorption”, “plastic”, “water”, and “soil” (2021–2022) underscore the shift towards investigating MP adsorption in aquatic environments and its influence on microbial communities in soil.

Three clusters dominate the analysis, characterized by the highest frequency and centrality metrics, namely, “remediation,” “plastic mulch,” and “microplastic”.

- Remediation: High-frequency keywords such as “soil”, “wastewater”, and “removal” dominated research from 2020 to 2022, reflecting efforts to mitigate MP pollution through biochar application in terrestrial and aquatic environments. From 2022 to 2024, terms like “community structure”, “agriculture”, and “impact” suggest a shift toward investigating biochar's influence on the physicochemical properties of soil and microbial community dynamics, particularly in agricultural systems.
- Plastic mulch: Keywords such as “microbial community” and “water” reflect studies (2020–2022) focusing on MPs derived from agricultural plastic mulch and their effects on microbial ecosystems and runoff. The research focus has since expanded (2022–2024) to encompass agricultural runoff assessments and urease enzyme activity in agricultural soils.
- Microplastics: “Biochar” remains the most central keyword, reinforcing its critical role in addressing MP contamination. From 2022 to 2024, the singular emergence of “nanoparticle” suggests a heightened research focus on biochar's ability to remove nanoscale MPs, given their significant risks to human health. However, detecting nanoplastics (NPs) in environmental matrices remains a challenge, necessitating advanced analytical techniques [30].

Additional keywords emerging from 2022 to 2024, such as “adsorbent”, “adsorbent mechanism”, “waste”, and “walnut shell”, highlight the development of biochar from agricultural residues and adsorption mechanism evaluations. Similarly, terms like “aggregate”, ”soil remediation”, “identification”, “mechanism”, and “kinetics” indicate a research trajectory focused on optimizing modified biochar materials for MP mitigation.

Overall, biochar-based MP remediation has been investigated in two principal environmental matrices, that is, aqueous systems and soil, with an increasing emphasis on agricultural soils and runoff. Research efforts are intensifying towards nanoscale MP removal due to their hazardous implications. However, NP detection remains complex, requiring state-of-the-art methodologies [30]. For soil matrices, studies aim to enhance soil properties, enzyme activity and beneficial microbial communities, ultimately improving crop productivity. This underscores the necessity of interdisciplinary research integrating material science, agriculture, and microbiology in biochar–MP–plant interactions.

Three primary research trajectories emerge from the keyword analysis:

- Investigating how different biochar synthesis methods influence physicochemical properties, remediation efficiency, and environmental stability of MPs.
- Evaluating biochar’s potential for MP remediation in agricultural soils, focusing on improvements in soil properties, crop yield, gene expression, and microbial communities.
- Examining biochar’s adsorption mechanisms and MP removal efficiency in wastewater treatment applications.

### Biochar synthesis techniques and functional optimization

#### Biochar synthesis

**Figure 4:**
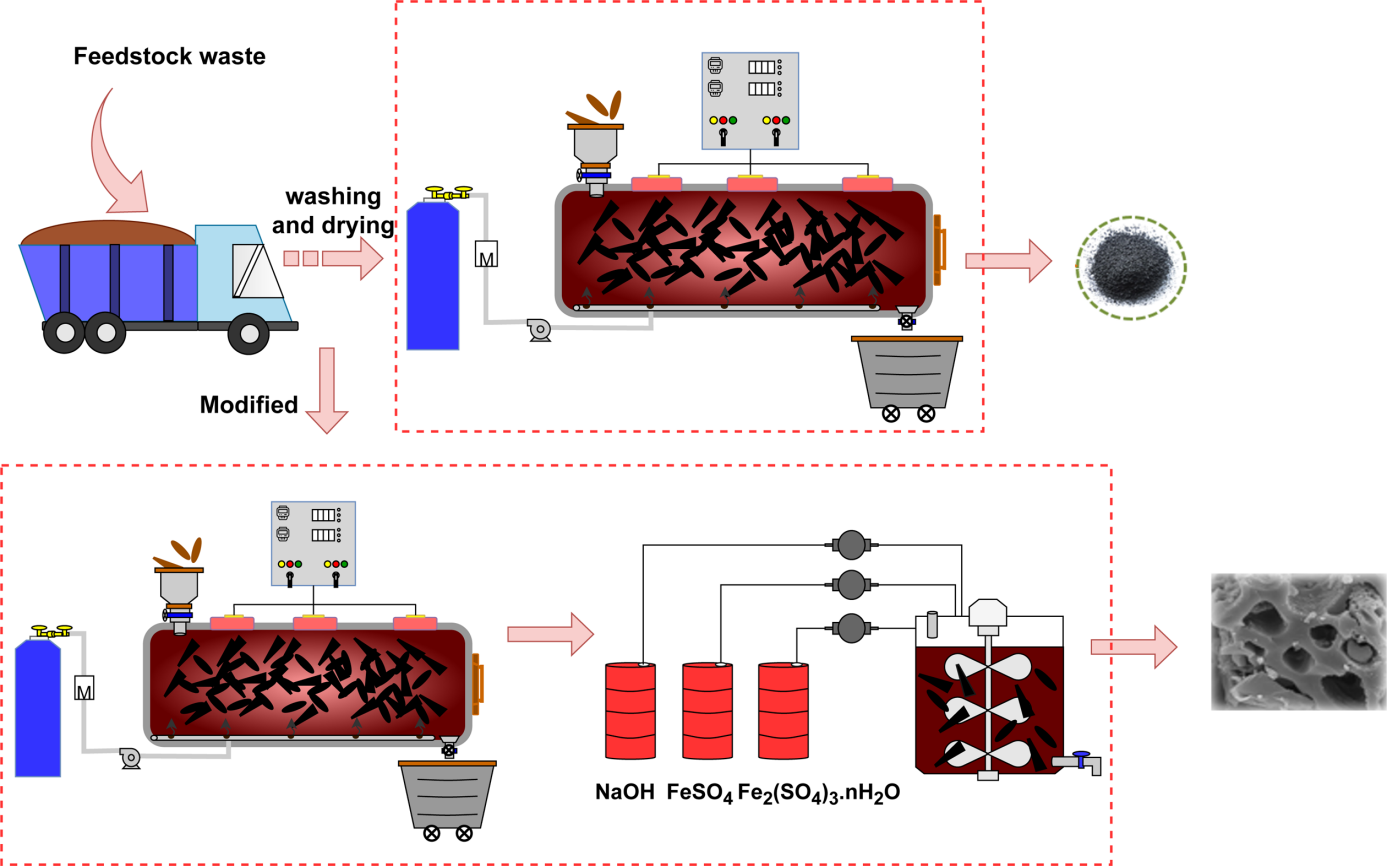
Production process of biochar and magnetic biochar.

The synthesis methods for biochar, as illustrated in Figure 4, encompass traditional hydrolysis techniques and modified approaches utilizing materials such as magnetic modifiers, magnetic-derived amphoteric metals and cooperative microbes [31–34]. Traditional biochar synthesis aims to optimize specific surface area and structural stability by controlling reaction time, heating rate and reactor temperature [35].

For example, biochar produced from oilseed rape straw and softwood pellets at 700 °C exhibited a carbon distribution and surface area 3.45–6.15 times greater than that obtained at 550 °C [36]. Similarly, biochar derived from straw feedstock showed an increase in specific surface area (SSA) from 37.2 to 302.8 m^2^·g^−1^ when the pyrolysis temperature increased from 300 to 800 °C [37]. However, [38] reported contrasting trends in commercial corn straw biochar, where increasing thermolysis temperatures led to a significant decrease in surface area from 808.3 to 177.5 m^2^·g^−1^, while the bulk density increased from 0.059 to 0.121 g·cm^−3^. Similarly, biochar derived from hardwood using an open-fire stove exhibited a relatively low surface area of 292.8 m^2^·g^−1^.

Modified biochar synthesis strategies aim to enhance adsorption capacity while maintaining stable pore volume. For instance, magnetic biochar (MBC) synthesized from peanut shells using co-precipitation exhibited increases in SSA from 10.25 to 205.46 m^2^·g^−1^ and pore volume from 0.02 to 0.3 cm^3^·g^−1^ [39]. Studies on bamboo-derived biochar indicated that SSA and pore volume generally increased with pyrolysis temperature, although the pore volume decreases above 700 °C, suggesting 700 °C as an optimal temperature [40]. Functional modifications, such as those incorporating hydrophilic functional groups, improve biochar’s ability to slowly release water molecules during dry cycles, reducing MP leaching risks and enhancing MP entrapment [41].

Temperature also affects functional groups in biochar. For example, enhanced diffusion, surface adsorption, and cation–π electron interactions were observed at 550 °C in *Polygonum amphibium L.* biochar. However, pyrolysis temperatures above 550 °C led to reduced stability [42]. Similarly, pyrolysis at 700 °C can increase SSA but may degrade oxygen-containing functional groups, affecting biochar stability [43].

The goal of modified biochar synthesis is to enhance its wastewater treatment and soil remediation capabilities by improving electrostatic and chemical bonding interactions with MPs, beyond the limitations of physical adsorption governed by specific surface area [44]. Magnetic biochar modification follows two main routes, that is, direct impregnation of feedstock or post-synthesis modification.

- Direct impregnation: Feedstock is washed and impregnated with 0.012 mol Fe(NO_3_)_3_·9H_2_O, stirred overnight, dried at 105 °C and heated at 550 °C for 2 h, yielding biochar with a surface area of 368.3 m^2^·g^−1^ for sawdust [32].
- Post-synthesis modification: Pre-synthesized biochar is immersed in ferric and ferrous solutions at pH 10–11 for 24 h, resulting in Fe_3_O_4_ deposition on the biochar surface [45].

The incorporation of functional groups significantly enhances the efficiency of BC in MP removal. Specifically, MBC derived from sludge and red mud has demonstrated improved adsorption capacity for nanoplastics [43]. Furthermore, modifying feedstock with urea, ascorbic acid, and iron salts before pyrolysis enhances iron and nitrogen content, improving microbial community interactions in soil [31].

#### Biochar properties

The physical and chemical properties of biochar depend significantly on the composition of its feedstock [46]. Molecular model calculations and quantum chemistry analyses suggest that biochar derived from wood exhibits superior physical properties, such as porosity and surface area, compared to other materials [37].

Biochar produced from carbon-, oxygen- and nitrogen-rich straw at low pyrolysis temperatures retains a large number of functional groups, which enhance charge transfer potential and adsorption stability through increased surface charge density, charge distribution, and bonding orbital characteristics [37]. Feedstocks and preparation conditions for biochar are listed in Table S1, Supporting Information File 1. Biochar derived from palm kernel shells and coconut shells showed lower carbon content than the corresponding raw material [47]. The O/C ratio indicates the stability of biochar, while the H/C ratio reflects the presence of fused aromatic hydrocarbons [48]. Most biochar samples in Table S2 (Supporting Information File 1) exhibit an H/C ratio below 0.15, indicating high aromatic hydrocarbon content, and an O/C ratio below 0.6, demonstrating high stability, particularly for biochar derived from agricultural byproducts. Carbon and nitrogen content in biochar are critical for improving soil nutrients and microbial populations. Comparisons of biochar and urea application revealed significant increases in total nitrogen content in soil, that is, 0.32 g/kg for 3% biochar addition and 0.36 g/kg for 3% biochar combined with urea [49]. Biochar amendments consistently enhanced nitrogen content in both topsoil (0–20 cm) and subsoil (20–40 cm) compared to urea [50]. These favorable properties highlight biochar’s potential for microplastic removal in water and its ability to restore functionality in microplastic-contaminated soils.

### Biochar applications in restoring MP-contaminated soil

#### Improving plant growth under MP stress

MPs negatively impact crop performance by reducing biomass production, inhibiting stem and root development and consequently affecting fruit and seed formation [51]. This effect is primarily due to oxidative stress and cellular damage in plant roots, which diminishes water and nutrient absorption [52]. MPs disrupt root–soil hydrocarbon exchange pathways and hinder photosynthesis. Furthermore, they impair metabolic processes such as the tricarboxylic acid cycle and galactose metabolism [53]. For instance, high rubber-MP dosages (10% MPs) led to significant reductions in both shoot and root biomass in peanuts after 48 days of growth [52]. Similarly, adding 1% polypropylene (PP) to soil during chili cultivation reduced root biomass, shoot biomass, and plant height compared to controls without PP contamination [51]. The biomass of peanut roots and aboveground parts decreased by 28.45% and 16.45%, respectively, with 1.5% polystyrene (PS) contamination in the soil [53]. For sugarcane, polyethylene (PE) contamination resulted in biomass reductions of 4.2–8.6% for canes and leaves and 22.6–37.9% for roots, indicating that root systems are the most affected [54].

**Figure 5:**
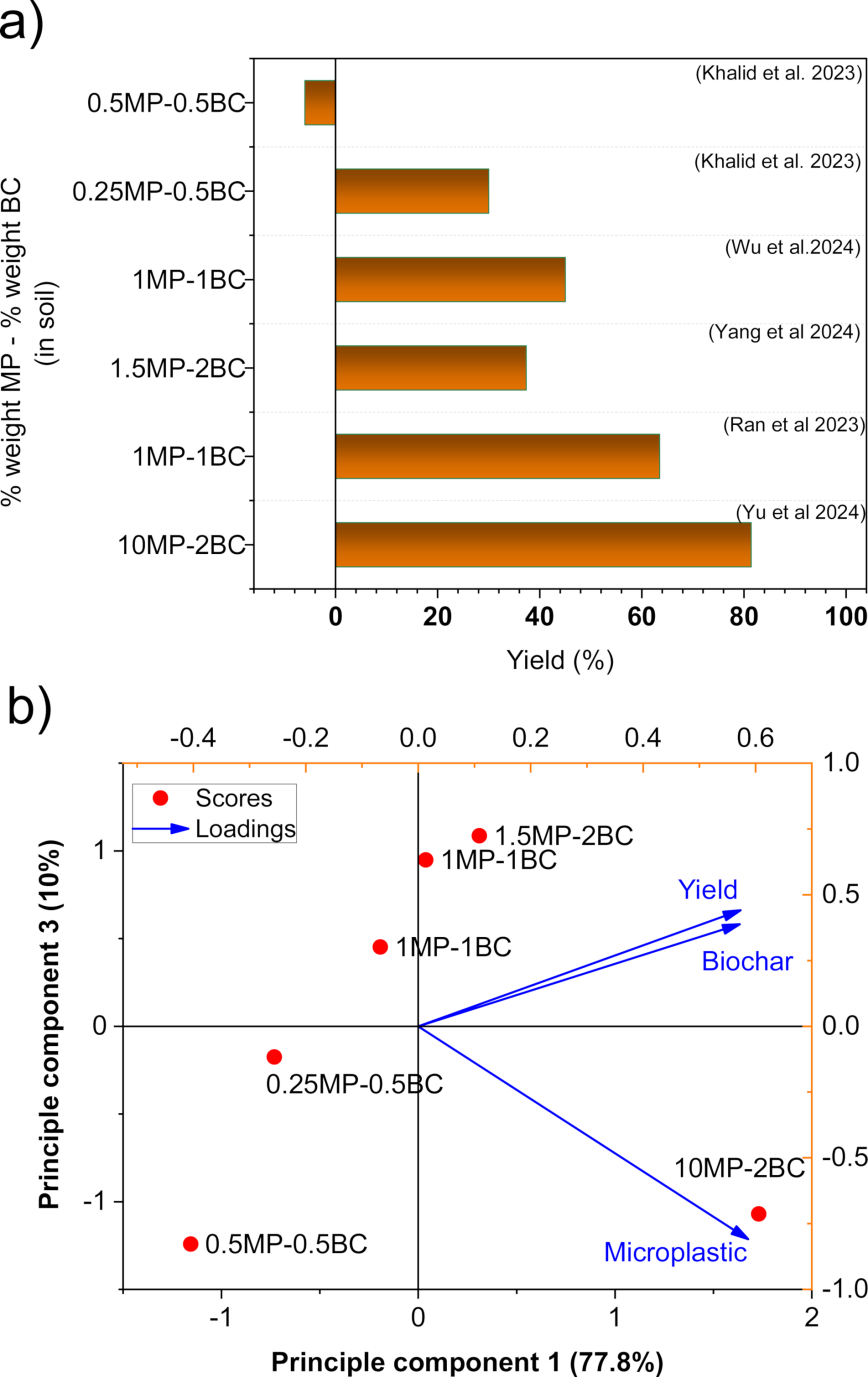
Plant biomass enhancement based on the percentage of MPs and BC in the soil (a) and PCA analysis based on yield, BC distribution and MPs (b).

The potential of biochar in mitigating MP-induced stress and enhancing plant biomass is illustrated in Figure 5. Experiments with 0.5% cotton stalk biochar in polyvinyl chloride (PVC)-contaminated soils during wheat cultivation showed improved shoot and root biomass at a PVC concentration of 0.25%. However, at higher PVC levels (0.5%), root recovery was less pronounced [55]. Based on the compiled data in Figure 5a, biochar supplementation at 1.0–2.0% resulted in more than 39% biomass increase. Specifically, applying 1% biochar enhanced biomass by 63.47%, 51.24%, and 92.20% during the seeding, flowering and fruiting stages, respectively [51].

Modified biochar, particularly when combined with beneficial microorganisms, exhibits even greater potential. For example, 1% corn stalk biochar-based microbial consortia (e.g., *Bacillus B28*) applied to soil containing 10% rubber crumb significantly increased peanut production by 58.86% and yield by 81.41% [52]. Principal component analysis (PCA) in Figure 5b reveals a strong correlation between biomass performance and biochar concentration, underscoring biochar’s pivotal role in soil remediation and plant performance enhancement in MP-contaminated soils.

#### Enhancement of soil physicochemical properties

MPs exert significant impacts on soil physicochemical properties, altering its physical structure, chemical composition, and microbial community. Regarding soil physical properties, MPs modify porosity and aeration, which in turn disrupt water and air circulation, leading to soil compaction and dryness that adversely affect root development [52,54,56]. These changes are exemplified by the reduction in water-stable aggregates by approximately 20% and a decline in saturated hydraulic conductivity by up to 70%. Moreover, the water content in soil at field capacity decreases between 10% and 65%, depending on soil texture, MP concentration, and size distribution [56].

Biochar application has shown potential in mitigating these adverse effects. For instance, adding 1.5% apple tree branch biochar to soil maintains the stability of large macroaggregates (>5 mm) and increases the soil organic carbon content in microaggregates by 35.7%, thus promoting the formation of microaggregates within macroaggregates [57]. This illustrates the effectiveness of biochar in counteracting the physical degradation caused by MPs.

In terms of soil chemical properties, MPs influence pH, organic matter content, and macronutrient levels, including nitrogen (N), phosphorus (P) and potassium (K). For example, 3–7% low-density polyethylene (LDPE) MPs significantly reduce total exchangeable cations (Na^+^, K^+^, Mg^2+^, and Ca^2+^) by 4–6% [58]. Additionally, non-biodegradable MPs such as PE and PVC inhibit organic phosphate mineralization, alkaline phosphatase activity, and inorganic phosphate solubilization. By contrast, biodegradable MPs such as PLA do not affect alkaline phosphatase activity [59]. MPs also reduce available phosphorus (Olsen-P) levels by 9.7–38.6% (PE, PVC) and 38.4–73.6% polyactide (PLA) [60]. Furthermore, MPs compete with plant roots for phosphate adsorption, with distribution coefficients (*K*_d_) in the order PVC (5.19 L/kg) > PE (4.23 L/kg) > PLA (2.48 L/kg) [60].

Biochar amendments can alleviate these chemical imbalances. For example, cotton and corn biochar added to soil contaminated with aged plastic mulch can increase Olsen-P levels by up to 46.6% during cotton cultivation [61]. These findings demonstrate the dual role of biochar in enhancing both physical and chemical properties of MP-contaminated soil, thereby improving overall soil functionality.

#### Enhancement of soil microbial communities

The restoration of soil nutrient cycling functions involves not only improving physicochemical properties but also addressing changes in biogeochemical cycles mediated by soil microbial communities. The effects of biochar on microbial diversity and activity in soils contaminated with MPs are summarized in Table S1, Supporting Information File 1. Biochar has demonstrated a capacity to enhance microbial diversity, as evidenced by multiple studies [51,53–54]. These improvements are most pronounced under well-watered conditions, although the effects on diversity may diminish or even reverse under dry or drought conditions [36].

Biochar also alters the composition of soil microbial communities exposed to MPs. Specifically, the abundance of microbial groups such as *Proteobacteria, Firmicutes, Patescibacteria* and *Cyanobacteria* decreases, while populations of *Actinobacteria, Chloroflexi, Acidobacteriota,* and chemoheterotrophs increase in the presence of MPs like PE, LDPE, and PS [53,62–63]. For instance, in wheat-cultivated soil exposed to PVC, biochar produced from cotton stalks at 650–750 °C increased microbial biomass nitrogen by 7–30% and microbial biomass carbon by 10–13%, enhancing the carbon and nitrogen cycling processes [55]. Similarly, the application of corn straw biochar in pepper-cultivated soil contaminated with PP elevated the abundance of *Acidobacteria and Bacteroidetes* by 1.32% and 1.37%, respectively [51]. Additionally, magnetic biochar restored fungal gene abundance in soils where PVC contamination (0.25% and 0.5%) had caused declines of 35.15% and 41.67%, respectively [55].

MPs are known to increase the abundance of antibiotic resistance genes (ARGs) in soil through adsorption, mobility, and propagation mechanisms [62]. MPs facilitate the movement and spread of ARGs by serving as carriers. For example, PS contamination at 1% increased ARG abundance to 0.26 copies per cell compared to 0.21 copies per cell in control soil [64]. MPs also promote the proliferation of multihost bacterial populations such as *Sphingomonas, Microvirga, Ilumatobacter, Skermanella,* and *Rubellimicrobium*, which harbor diverse ARGs. Increased populations of *Bacillus* and *Streptomyces* have also been associated with PS exposure [62,64]. Furthermore, the addition of fungicides to MP-contaminated soil has been linked to increased resistance genes for rifamycin, vancomycin, novobiocin, quinolone, and mupirocin [65]. However, studies have shown that coconut shell biochar can reduce ARGs, including sulfonamide and tetracycline resistance genes, by up to 88.57% and mobile genetic elements by 48.33–56.72% [62].

In addition to reducing ARGs, biochar positively impacts beneficial soil microbial populations and enzymatic activities, contributing to soil functionality. Biochar enhances the abundance of beneficial microbes such as *Subgroup 10*, *Bacillus* and *Pseudomonas*, which suppress harmful bacteria and promote plant growth [54]. For instance, in lettuce rhizosphere soil exposed to PS, genetic diversity decreased by 26.67%, but the application of peanut shell biochar increased gene abundance by 5.15% compared to control soil [53]. Moreover, biochar integrated with microbial consortia has been shown to increase urease and dehydrogenase activities by 19.65% and 115.74%, respectively, in rubber crumb-contaminated soil [52]. Similar trends were observed with cotton stalk biochar in PVC-contaminated soils, although dehydrogenase activity increased only by 5–15%, indicating lower effectiveness compared to biochar integrated with microbial consortia [55]. Conversely, biochar derived from food waste showed a tendency to reduce urease and fluorescein diacetate activities, likely due to the heterogeneous composition of food waste, which results in inconsistent biochar properties and enzymatic effects [63].

Recent studies conducted between 2022 and 2024 on BC amendment in MP-contaminated soils have underscored its critical role in mitigating the adverse effects of MPs on soil ecosystems. BC facilitates these improvements by interacting with plants, activating genes associated with oxidative stress resistance, enhancing soil properties and promoting the growth of beneficial microorganisms, enzyme activity, and ARGs. Through these mechanisms, BC contributes to restoring microbial equilibrium, regulating enzymatic functions and modulating plant gene expression and ARG dynamics in the soil. Ultimately, these effects foster a healthier and more resilient soil ecosystem under MP contamination.

### Water treatment

#### MP removal from aqueous media by BC adsorption

A summary of research on MP removal is provided in Table S2, Supporting Information File 1, covering a range of MPs, including PE, PS, polyamide (PA), and mixtures such as LDPE, polyethylene terephthalate (PET), PA, polycarbonate, PE, PS, and PVC [33,42,66–67]. Among these, PS is frequently utilized as a model MP due to its uniform composition and quantifiability, making it a reliable candidate for modeling and experimental investigations [33]. According to Shams et al. [68], PS exhibits a critical coagulation concentration of 800 mM NaCl, significantly higher than PE’s 80 mM, which underscores PS’s stability in saline environments and its suitability for long-term studies. The size of MPs is a critical factor regarding human health risks. MPs ranging from 40 to 4819 µm are typically excreted through human feces; however, MPs sized 20–103 µm can enter body fluids, and those averaging at 9.8 µm can infiltrate the liver. Even smaller MPs, ranging from 1 to 469 µm, can penetrate the heart and kidneys [69]. Therefore, the removal of increasingly smaller MPs has become an urgent focus in MP treatment research.

The treatment of PS using biochar derived from London plane tree pyrolyzed at 550 °C in a complete mixing model demonstrated an adsorption capacity of 60.05 mg·g^−1^, adhering to second-order reaction kinetics [42]. In another approach, filtration through biochar derived from banana peel achieved a removal efficiency of up to 92.16% for PS particles sized 150–300 µm [67]. An even higher removal efficiency, reaching 99%, was observed in a dual-layer filtration model utilizing silica sand and corn straw biochar for 10 µm PS particles [38]. For particles larger than 75 µm, mechanisms such as hydrogen bonding and hydrophobic interactions dominate, while smaller particles (<75 µm) are removed through π–π interactions between the benzene rings of PS and biochar surfaces, surface adsorption, hydrogen bonding, and surface complexation [67]. These findings highlight the potential of diverse biochar configurations for efficient MP removal under various conditions. Recent studies on biochar derived from coffee waste modified with amino-functionalized zeolite/phosphoric acid (AZP) highlight its potential for removing PS particles as small as 6 µm, with adsorption capacities ranging from 4.78 to 4.85 mg·g^−1^ [34]. Some studies have also reported MP removal for sizes below 1 µm. Specifically, PS particles ranging from 1 to 1000 nm treated with MBC achieved a maximum removal efficiency of 40.93% [39]. However, the study by Feng, et al. [43] reported that MBC derived from red mud mixed with lignin exhibited a removal efficiency for 100 nm PS particles ranging from 56% to 97.87%, depending on the MBC/PS ratio (0.01–10).

Magnetic biochar and Zn-modified magnetic biochar (MBC/MBC-Zn) further enhance removal through electrostatic interactions, forming metal–O–PS–MP bonds. These advanced materials demonstrated removal efficiencies of 96.24% and 84.77%, respectively, outperforming traditional biochar models like banana peel biochar, which achieved a removal rate of 96.5% for PS at a concentration of 0.2 g/L [70]. The integration of such mechanisms underscores the effectiveness of MBC and MBC-Zn in treating high-concentration PS systems.

For PE, current studies reveal high removal efficiencies of about 92–94% for both large microbeads (2–3 mm) and fine particles (10 µm) by BC [47]. For instance, Siipola, et al. [71] demonstrated near-complete removal of PE particles in fixed-column models using Scots pine bark and spruce bark, with adsorbent surface areas ranging from 187 to 603 m^2^·g^−1^. Similarly, filtration models employing palm kernel shell and coconut shell biochar showed performance highly dependent on biochar particle size and column dimensions [47]. Specifically, biochar sized 0.6–1.18 mm combined with a column diameter of 15 mm and a 20 cm bed depth achieved up to 96.65% removal efficiency. For 10 µm PE particles, retention was limited to segments 3–9 (out of 29 segments) with optimal removal observed for adsorbents with surface areas of 539 m^2^·g^−1^ [71]. Biochar derived from jujube waste pyrolyzed at 700 °C achieved more than 99% removal efficiency for PE, compared to 98% for biochar produced at 300 °C, at the optimal pH 7 [72]. Notably, PE removal efficiency was lower than that for nylon, particularly in pore volumes ranging from 1 to 11, due to differences in charge and surface interactions.

**Figure 6:**
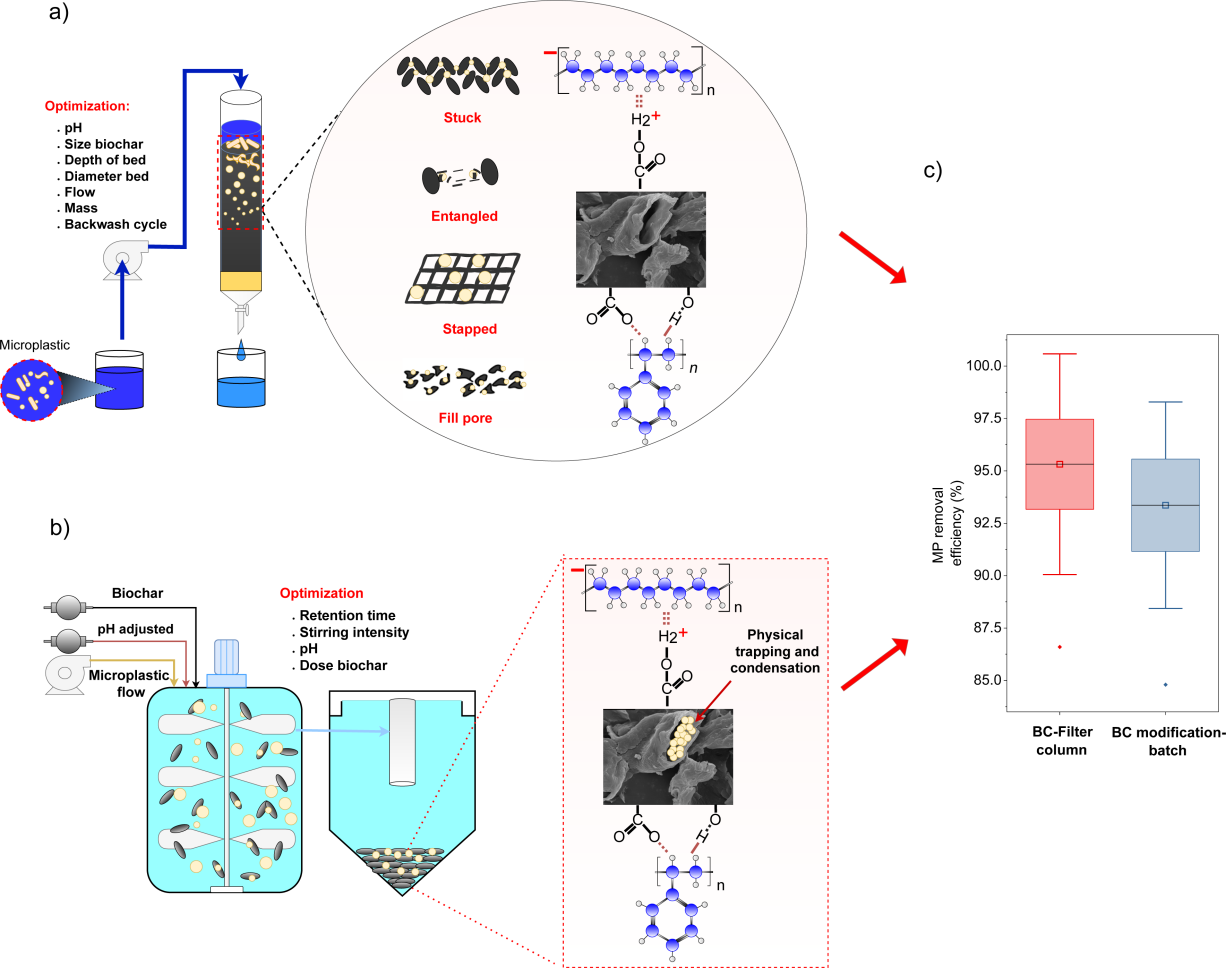
Mechanism of microplastic removal using biochar in a column filtration model.

Conventional MP treatment models using BC include batch tanks and filter columns, as presented in Table S3, Supporting Information File 1. Based on statistical results depicted in Figure 6c, the MP removal efficiency using filter column models is 95.31% ± 5.26%, while the batch-modified BC model achieved 93.36% ± 4.92%. Fixed columns are highly effective for MP sizes of approximately 10 µm and larger, whereas batch methods are preferred for MPs smaller than 10 µm. The efficiencies of these two models are based on different mechanisms, as described in Figure 6. MP removal by BC filter columns is primarily facilitated by the fixation of particles through filtration effects, entanglement due to surface roughness and hydrophobic interactions [47].

The negatively charged surfaces of both biochar and PE interact via van der Waals forces, whereas positively charged nylon particles exhibit higher removal efficiency due to electrostatic π–π interactions with biochar [66]. The efficiency of batch-based MP removal is significantly influenced by the physicochemical properties of biochar, MP particle size and the aggregation potential of MPs [43,67]. These findings underscore the importance of optimizing biochar properties to enhance MP removal across different particle types and sizes. Despite advancements in biochar-based filtration and batch treatment, the removal efficiency for nanoplastics remains suboptimal. For instance, the adsorption capacity of biochar derived from London Plane bark for nanostyrene is limited to 60.05 mg·g^−1^, while peanut shell biochar achieves only 16.47% removal efficiency [39,42]. However, modifications such as magnetic enhancement have improved filtration efficiency, with MBC reaching 40.86% removal. Moreover, MBC synthesized from red mud and lignin composites exhibits significantly enhanced adsorption performance, achieving 97.87% removal efficiency and a maximum adsorption capacity (*q*_max_) of 353.15 mg·g^−1^ for nano-PS (100 nm) [39,43]. These findings indicate that while different treatment models exhibit varying efficiency, the removal of nanoscale MPs predominantly depends on functional group interactions within biochar.

The presence of functional groups in AZP such as C–H, C–O, C=C, N–H, Al–O, and Si–O, along with its graphite-like structure, facilitates enhanced physical and chemical adsorption, as well as electrostatic interactions, thereby improving the removal of small MP particles [34]. Additionally, PS adsorption mechanisms suggest that MP self-polymerization and the strengthened metal–O bonding in nano-iron-modified biochar contribute to increased MP removal efficiency, particularly for nanosized particles [43]. Field studies on agricultural runoff reveal diverse MP contamination, including PVC films (500–1000 µm), fragments (45–500 µm, comprising PS, PA, PC, HDPE, LDPE, PET), and beads (40–48 µm, consisting solely of PE). Treatment using biochar derived from sugarcane achieved an overall removal efficiency of 92.6%, with the lowest performance observed for PET > PE > PA > ABS [66]. Hsieh, et al. [73] reported that PVC-contaminated water containing particles smaller than 10 µm could be effectively treated using a fixed-bed column. The filtration system employed sand as the primary medium, amended with 1% woodchip biochar produced at 700 °C, resulting in a removal efficiency of 92.91%.

Between 2022 and 2024, research on MP removal via biochar has increasingly focused on modified biochar to enhance the adsorption of small MP particles. These studies contribute to a deeper understanding of adsorption mechanisms based on biochar material properties. While these findings demonstrate significant progress in MP treatment, they also highlight the limitations of current research, which primarily focuses on single-component MP removal. Future efforts should emphasize developing tailored biochar materials for challenging MPs, such as PET and ABS, and addressing smaller particles below 10 µm to ensure comprehensive treatment in diverse environmental contexts.

#### Adsorption kinetics of MPs on biochar

Adsorption kinetics provide insights into the rate at which MPs adhere to biochar surfaces. Most studies employ pseudo-first-order (PFO) and pseudo-second-order (PSO) models to describe adsorption behavior [74]. The analysis of kinetic models for both biochar and modified biochar reveals that the correlation coefficient (*R*^2^) for PSO is consistently higher than that for PFO, indicating that MP adsorption on biochar is primarily governed by chemisorption processes [75]. For instance, MP removal using MBC derived from red mud shows low *R*^2^ values (0.14–0.23) for PFO but a significantly higher *R*^2^ (0.99) for PSO, demonstrating the crucial role of metal–O functional groups in adsorption [43]. In contrast, the adsorption kinetics of AZP show minimal differences between PFO and PSO models, suggesting a balanced contribution from its graphene-like structure and Al–O/Si–O functional groups. The mixed first- and second-order model achieves an *R*^2^ value of 1.0, further supporting this dual contribution [34]. Additionally, Li, et al. [33] reported that the Elovich model outperformed PSO, highlighting the role of heterogeneous surface interactions in MP adsorption onto biochar. The correlation analysis presented in Figure S2, Supporting Information File 1, demonstrates a strong inverse relationship between MP size and the second-order rate constant (*k*), with a coefficient of determination of 0.87 and a correlation coefficient of 0.93.

#### Comparison with other MP removal techniques

Various MP removal techniques in aqueous matrices, such as filtration, adsorption, and coagulation, have been extensively studied (Table S4, Supporting Information File 1). Among these, electrocoagulation demonstrates the highest removal efficiency (>98%) at an optimal pH of approximately 7 [76–77]. Although charcoal-based treatment for MPs smaller than 250 µm achieves a relatively high removal efficiency of 94.12%, its low adsorption capacity (4.5 mg·g^−1^) necessitates the use of a large quantity of adsorbent to ensure effective removal [78]. Notably, aluminum anodes have shown superior performance compared to iron anodes, with removal efficiencies, varying according to MP type and size, of 98.4% for PP (1–2 mm), 98.2% for cellulose acetate (1–2 mm), 91.7% for PE (286.7 µm), and 93.2% for polymethyl methacrylate (6.3 µm) [77]. The dielectric properties of MPs also influence electrocoagulation performance; for example, PVC (insulator 10 × 10^5^ Ω·cm) undergoes optimal treatment faster than PS (insulator 16 × 10^5^ Ω·cm). Membrane filtration using PVDF in lab-scale experiments achieves 100% efficiency at a pressure of 2 bar; however, in industrial settings, efficiency drops to 54.6% due to high-pressure requirements [76]. Membrane fouling remains a major drawback, with ultrafiltration showing lower flux recovery compared to membrane filtration [79]. Biocoagulation using *Chlorella vulgaris* achieves a maximum removal efficiency of 73.01% for PS (65.49–328.4 µm), while *Spirulina platensis* reaches 81% for PS (328.4 µm) [80–81]. *Abelmoschus esculentus*-based biocoagulation achieves 64.46% and 80.11% efficiencies for PS and PVC (<100 µm), respectively [82]. While biochar and biocoagulation methods are environmentally friendly, their efficiency remains limited, particularly for MPs <10 µm. Furthermore, no studies have addressed the separation of biomass from MPs, whereas MP detachment from biochar is feasible during regeneration, allowing biochar to be reused effectively.

Electrocoagulation, despite its high MP removal efficiency and effectiveness for particles smaller than 10 µm, presents environmental challenges due to the production of metal-laden sludge. In contrast, biochar offers an environmentally sustainable alternative, leveraging agricultural waste while allowing for MP separation and biochar regeneration. These attributes highlight biochar's potential as a viable solution for MP remediation in diverse environmental contexts.

#### Practical implications and policy relevance

Bibliometric analysis serves as a useful tool for mapping research trends; however, its scope is constrained by its reliance on indexed publications within databases such as WOS, thereby limiting the comprehensiveness of the assessment [83–84]. This method primarily indexes citations and frequently referenced topics, providing macroscopic visualization rather than in-depth insights into specific research domains. Additionally, despite the support of specialized bibliometric software, the potential for subjective biases remains a concern, necessitating a critical and contextualized interpretation of results [85]. A deep and systematic understanding of the field is therefore essential to ensure precise evaluations.

Biochar and modified biochar have been widely recognized as promising materials for microplastic removal; however, their application at pilot or full-scale levels has not yet been extensively studied. As a result, uncertainties persist regarding the actual removal efficiency of these materials under real-world conditions. Furthermore, critical factors such as production costs and quality consistency have not been adequately addressed in current research. Existing policies related to microplastics predominantly emphasize plastic waste reduction and recycling, without establishing clear regulatory thresholds for microplastic concentrations in water and soil [86]. Given the well-documented environmental and biological hazards posed by microplastic pollution, biochar’s multifunctionality presents a promising approach for mitigation. Nonetheless, further research is required to bridge the gap between laboratory-scale studies and real-world applications, ensuring technological feasibility, economic viability, and alignment with future regulatory frameworks.

## Conclusion and Future Perspectives

This paper provides a comprehensive overview of global research trends and recent progress in MP removal using biochar, based on a bibliometric analysis of relevant literature. The analysis highlights biochar as a promising solution, deserving extensive attention. The co-citation analysis and cluster views reveal the importance of interdisciplinary cooperation, suggesting that involving researchers from diverse scientific fields can yield valuable insights into tackling MP pollution. Despite its potential, significant practical challenges remain, necessitating further research to optimize biochar’s application.

Biochar has demonstrated remarkable potential in mitigating the impacts of MPs on soil and plants. It enhances plant biomass yield by up to 80%, improves soil water retention and cation exchange capacity and increases Olsen-P levels. It also fosters the growth of beneficial soil bacteria, such as *Subgroup 10*, *Bacillus*, and *Pseudomonas*, which suppress harmful microorganisms, reduce antibiotic resistance genes and enhance biodiversity. The increased activity of soil enzymes, including urease and dehydrogenase, further illustrates its role in improving soil fertility. Notably, microbial-enriched biochar exhibits the highest enzymatic activity, presenting significant opportunities for application in soil remediation.

In experimental setups for MP removal, biochar has proven highly effective. Modified biochars, such as magnetic biochar and Zn-modified biochar, enhance electrostatic interactions with MPs, achieving removal efficiencies ranging from 78% to 99%. Advanced biochar materials, including amino-functionalized zeolite series, have demonstrated the ability to remove MPs as small as 6 µm in batch systems. Promising results have also been obtained for nanoplastic removal using magnetic biochar, further demonstrating its adaptability as a remediation tool.

Despite significant progress, several critical challenges must be addressed before biochar can be widely applied for MP remediation. Optimization of surface properties remains essential to enhance removal efficiency, particularly for smaller MPs. The scalability of biochar applications requires further validation through pilot studies and field-scale implementation. Additionally, the integration of biochar with advanced treatment technologies, such as fluidized-bed systems, could enhance process feasibility.

Regulatory frameworks for MP pollution remain underdeveloped, necessitating standardized guidelines for permissible concentrations in environmental matrices. Evidence-based policies are required to support large-scale adoption while ensuring the safety and efficacy of biochar-based remediation strategies. The development of microbial-enriched biochar presents new opportunities for soil restoration, yet further research is needed to optimize its composition and stability.

Advancements in computational modeling, including machine learning and experimental design methodologies, could facilitate the optimization of biochar performance. The economic feasibility of biochar production remains a major concern, requiring cost-reduction strategies to enhance its practical applicability. Additionally, the potential release of contaminants during biochar synthesis and application must be carefully assessed to mitigate unintended environmental risks.

Future research should focus on addressing these challenges through interdisciplinary collaboration, ensuring that biochar-based approaches contribute to sustainable and effective MP mitigation strategies.

## Supporting Information

File 1 Additional data.

## Data Availability

All data that supports the findings of this study is available in the published article and/or the supporting information of this article. Additional data can be made available upon reasonable request.
