# Supplementary material for: The role of biochar in combating microplastic pollution: a bibliometric analysis in environmental contexts
Source: Beilstein J Nanotechnol. 2025 Aug 21;16:1401–16. doi: 10.3762/bjnano.16.102 (PMC12415920; doi:10.3762/bjnano.16.102)
Supplement: File 1 — Additional data. [file Beilstein_J_Nanotechnol-16-1401-s001.pdf]

BEILSTEIN JOURNAL OF NANOTECHNOLOGY

Supporting Information

for

The role of biochar in combating microplastic pollution: a bibliometric analysis in environmental contexts

Tuan Minh Truong Dang, Thao Thu Thi Huynh, Guo-Ping Chang-Chien and Ha Manh Bui

Beilstein J. Nanotechnol. **2025,** *16,* 1401–1416. doi:10.3762/bjnano.16.102

Additional data

License and Terms: This is a supporting information file under the terms of the Creative Commons Attribution License (https://creativecommons.org/licenses/by/4.0). Please note that the reuse, redistribution and reproduction in particular requires that the author(s) and source are credited and that individual graphics may be subject to special legal provisions.
The license is subject to the *Beilstein Journal of Nanotechnology* terms and conditions: (https://www.beilstein-journals.org/bjnano/terms)

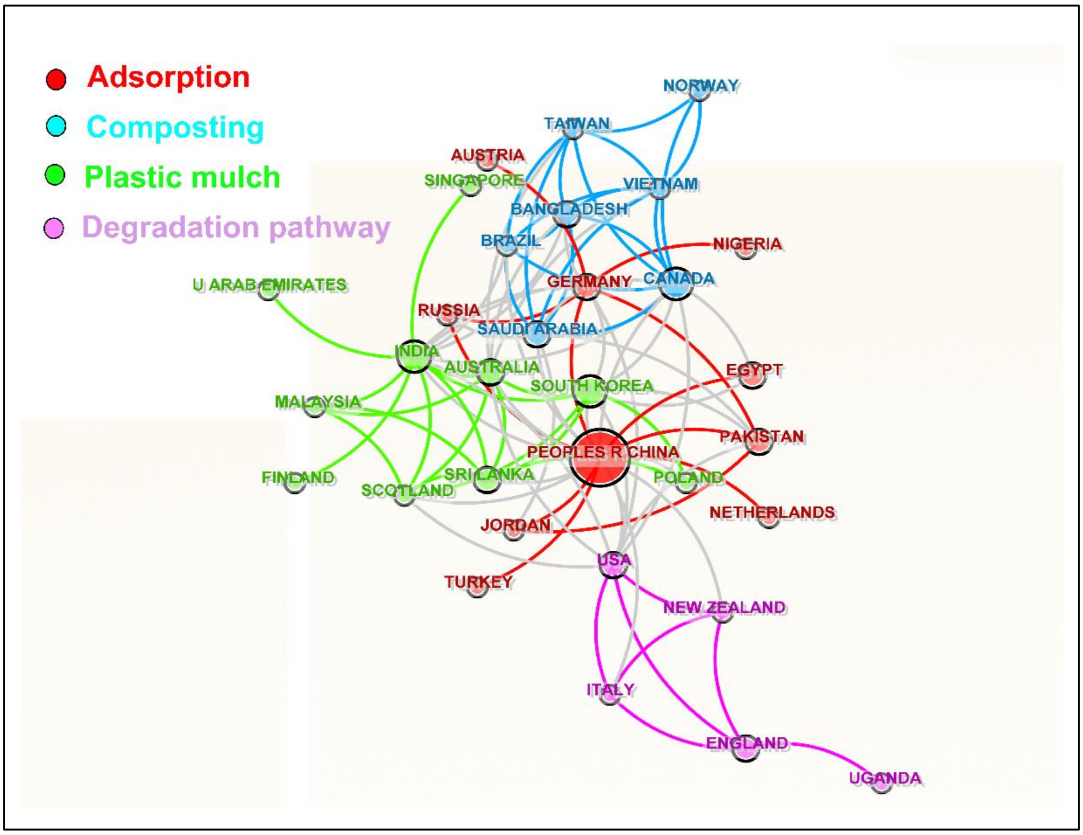

Figure S1: Co-occurrence of regional cooperation networks.

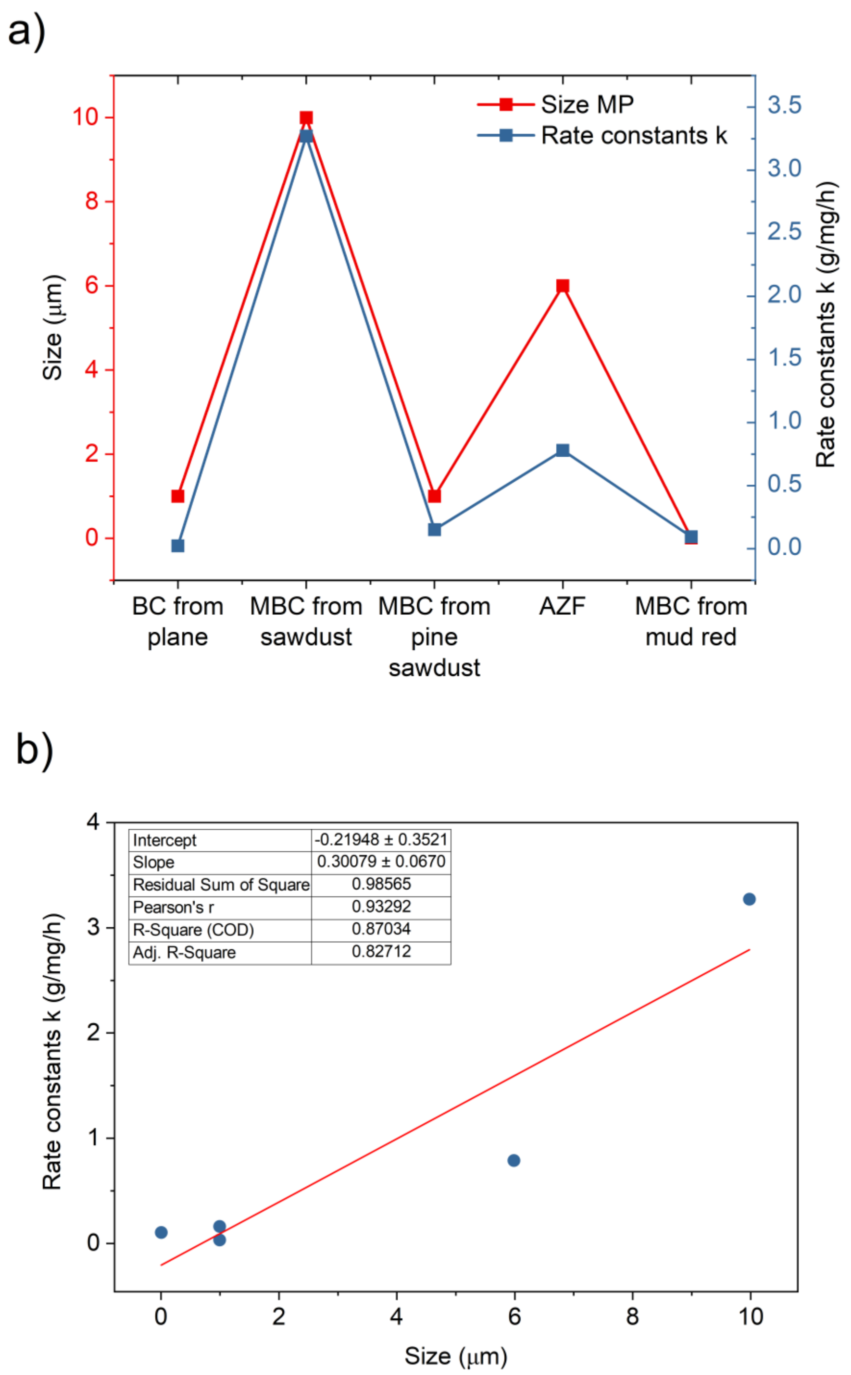

Figure S2: Rate constants for the adsorption kinetics of BC on micro PS (a) and the regression equation based on the rate constant and the size of micro PS (b).

Table S1: Biochar characteristics from different feedstocks.

Biochar	Temperature (°C)/time	pH	C (wt%)	H (wt%)	N (wt%)	O (wt%)	Fe (wt%)	S (wt%)	P (wt%)	H/C	O/C	Specific surface area (m^2 g^{-1})	Pore volume (cm^3 g^{-1})	References
**Soil**														
BC from rice straw	700 °C, 2 h	-	52.01	1.05	0.57	17.87	-	-	0.24	0.02	0.26	106.41	-	[1]
MEBC-from rice straw	• Heated for 10 h at 160 °C<br>• pyrolyzed at 700 °C under N_2 gas for 2 h	-	18.47	-	2.11	5.96	30.37	33.56	-	-	0.24	120.84	-	
OSR	550 °C	9.78	68.85	1.82	1.29	8.91	-	-	0.29	0.32	0.1	7.3		[2]
	700 °C	10.41	67.74	1.09	1.26	7.84	-	-	0.26	0.19	0.09	25.2		
SWP	550 °C	7.91	85.52	2.77	<0.1	10.36	-	-	0.06	0.39	0.09	26.4		
	700 °C	8.44	90.21	1.83	<0.1	6.02	-	-	0.07	0.24	0.05	162.3		
BC from peanut shells*	450 °C, 4 h	8.85	678.45	-	7.73	-	-	-	1.2	-	-	38.42	0.024	[3]

Biochar	Temperature (°C)/time	pH	C (wt%)	H (wt%)	N (wt%)	O (wt%)	Fe (wt%)	S (wt%)	P (wt%)	H/C	O/C	Specific surface area (m^2 g^{-1})	Pore volume (cm^3 g^{-1})	References
Bulk BC from coconut shell	Commercial	-	78.75	2.16	0.63	-	-	-	-	-	-	902.64	0.26	[4]
Nano BC from coconut shell		-	94.2	-	0.47	-	-	-	-	-	-	77.35	2.65 x10^{-3}	
BC from food waste	500 °C, 20 min	9.10	55.87	6.5	4.49	-	-	-	-	0.116	-	-	-	[5]
BC from rubber crumb	450 °C, 2h	-	62.49	2.46	2.57	18.08	-	-	-	0.04	0.29	108.02	0.1 (Include medium pore and micropore)	[6]
Corn straw	650–750 °C	9.6	54.5	0.7	0.79	0.79	4.4	-	1.8	0.013	0.014	-	-	[7]

Biochar	Temperature (°C)/time	pH	C (wt%)	H (wt%)	N (wt%)	O (wt%)	Fe (wt%)	S (wt%)	P (wt%)	H/C	O/C	Specific surface area (m^2 g^{-1})	Pore volume (cm^3 g^{-1})	References
Corn straw*	500 °C for 2 to 3 h	7.29	55.31	-	1.35	-	-	-	0.243	0.02	0.34	-	-	[8]
**Water**														
corn straw	300	-	70	-	3.1	18.7	-	0.2	1.2	-	0.27	808.3	-	[9]
	400	-	81.3	-	3.7	11.7	-	0.3	0.2	-	0.14	609.0	-	
	500	-	68.7	-	2.2	19.1	0.1	0.2	1.5	-	0.28	177.5	-	
Hardwood	Produced through a traditional open fire stove	-	84.7	-	0.3	14.1	-	-	-	-	0.166	292	-	
Palm kernel shell	600 °C for 4 h	-	63	1.61	0.24	2.79	-	<0.6	-	0.025	0.04	-	-	[10]
Coconut shell biochar		-	78	1.52	< 0.1	2.94	-	<0.6	-	0.019	0.037	-	-	

Biochar	Temperature (°C)/time	pH	C (wt%)	H (wt%)	N (wt%)	O (wt%)	Fe (wt%)	S (wt%)	P (wt%)	H/C	O/C	Specific surface area ($m^2 g^{-1}$)	Pore volume ($cm^3 g^{-1}$)	References
MBC from sawdust	550 °C (with a heating rate of 5 °C/min) for 2 h	-	69.29	-	-	20.38	10.33	-	-	-	0.22	363.8	0.37	[11]
Mg-MBC		-	65.03	-	-	16.93	11.20	-	-	-	0.20	265.47	0.41	
Zn-MBC	550 °C for 2.5 h at a heating rate of 30 °C min^{-1}	-	66.48	-	-	15	12.45	-	-	-	0.17	329.87	0.34	
Amino-Functionalized Zeolite Series/H_3PO_4	H_3PO_4 impregnation Co – pyrolysis waste coffee and ammonium ZSM-zeolite	-	-	-	-	-	-	-	-	-	-	79	0.09	[12]

Biochar	Temperature (°C)/time	pH	C (wt%)	H (wt%)	N (wt%)	O (wt%)	Fe (wt%)	S (wt%)	P (wt%)	H/C	O/C	Specific surface area (m^2 g^{-1})	Pore volume (cm^3 g^{-1})	References
Peanut shells	low pyrolysis at 500 °C for 2 h	8.7	57.35	2.22	1.47	5.44	-	-	-	0.46	0.09		0.02	[13]
MBC from peanut shells	prepared using the co-precipitation method	7.92	43.55	1.93	1.41	6.11	-	-	-	0.53	0.14	205.46	0.3	

Table S2: Biochar application for soil remediation and plant growth in MP-polluted environments.

Soil/ plant class	Biochar category	MPs parent	Efficiency (%)				Note	Reference
			Genes/Enzyme		Bacteria			
			Kind	*Tend	Kind	*Tend		
Soil layer in farmland	Bulk and biochar coconut shell biochar	PE	Aminoglycoside, sulfonamides, tetracycline multiple drug resistance, integrons, transposons	88.57 (Based on total of genes)	*Actinobacteriota, Proteobacteria, Chloroflexi, Acidobacteriota, Firmicutes*	Increased: *Actinobacteriota, Chloroflexi* and *Acidobacteriota*, Decreased: *Proteobacteria, Firmicutes* and *Patescibacteria*.	• Biochar only reduces antibiotic genes caused by MPs. • Nano-biochar has a better inhibitory effect than bulk biochar. • MPs increase multiple drug resistance genes.	[4]

Soil/ plant class	Biochar category	MPs parent	Efficiency (%)				Note	Reference
			Genes/Enzyme		Bacteria			
			Kind	*Tend	Kind	*Tend		
Agricultural field	Oilseed rape straw (ORS), Softwood pellets (SWP)	LDPE	Urease: 77.34 μg N hydrolyzed/ g	OSR: –<br>SWR-700:+	*Acidobacteria*	Increase with SWP-550 and SWP-700	• The difference in alkalinity and EC depends on raw materials.<br>• Larger pore volume allows easier development of urease microorganisms.	[2]
			Fluorescein diacetate: 1.69 μg FDA g ×min	OSR-700: +28.47%<br>SWP-700: + 30.71%	*Proteobacteria*	Decrease		
			Acid phosphatase	ORS-500: –<br>ORS-700: –	*Firmicutes*	Decrease (ORS-500, - 700 and SWP 700)		
Lettuce rhizosphere soil	Charring peanut shells	PS	1677 gen detected in Rhizosphere soil	MPs: Decrease 26.67%	*Chemoheterotrophy*	Increase 10% of the total	• Biochar reduces the inhibitory effects of	[3]
					Aerobic Chemoheterotrophy	Increase 5% of the total		

Soil/ plant class	Biochar category	MPs parent	Efficiency (%)				Note	Reference
			Genes/Enzyme		Bacteria			
			Kind	*Tend	Kind	*Tend		
				Biochar: Increase 5.15%	*Cyanobacteria, oxygenic photoautotrophy*	Decrease 90% of the total	microplastics on plant growth, especially root growth.<br>• Biochar positively affects genes related to antioxidation and nitrogen transport.	
Lettuce cultivated soil	Food waste-derived biochar	PS	Urease	– 46.2% (MPs and BC)	*Chloroflexi, Planctomycetes*	Highest distribution in total experiments surved BC	• PS plastic does not significantly affect soil chemical properties but	[5]
			Fluorescein diacetate	– 94% (MPs and BC)				

Soil/ plant class	Biochar category	MPs parent	Efficiency (%)				Note	Reference
			Genes/Enzyme		Bacteria			
			Kind	*Tend	Kind	*Tend		
			Acid phosphatase	+ 30% (MPs and BC)			causes pressure on microorganisms.<br>• BC enhances root-associated microbial communities and is a suitable choice.	
Peanuts cultivated soil	Corn stalks biochar inter integrated microbial	Tire-derived rubber crumbs	urease	+19.65%	*Pseudomonas, Sheathbacteria, Burkholderia* and *Solibacter*	Significant reduction when MP is added	• Changes in soil bacteria due to SynCom treatment can affect nitrogen cycling.<br>• RC-MP addition alters root	[6]
			dehydrogenase	+115.74%	*Pseudomonas*	Increase three times with BC		

Soil/ plant class	Biochar category	MPs parent	Efficiency (%)				Note	Reference
			Genes/Enzyme		Bacteria			
			Kind	*Tend	Kind	*Tend		
							bacterial populations, influencing peanut plant development.	
Agricultural wheat soil	Cotton stalks (650 – 750ºC)	PVC	urease	+ 14.45 – 26%	Microbio biomass nitrogen	↑ 7 – 30%	• PVC affects carbon distribution in plant stems and roots, leading to reduced yield. It also impacts Gram-positive bacteria and Pseudomonas.	[7]
			dehydrogenase	+ 5 – 15%	Microbio biomass carbon	+ 10%- 13%		

Soil/ plant class	Biochar category	MPs parent	Efficiency (%)				Note	Reference
			Genes/Enzyme		Bacteria			
			Kind	*Tend	Kind	*Tend		
							• Biochar increases urease activity even in the presence of PVC-MPs, reducing the toxic effects of MPs on rRNA 16S or 18S and bacterial diversity.	
Agricultural pepper soil	Corn straw at 500 °C	PP	Pielou index, Shannon index and Observed ASVs	Bacterial α Diversity Increased	*Acidobacteria*	+ 1.32% distribution	• *Proteobacteria, Chloroflexi, Acidobacteriota, Bacteroidota, Actinobacteriota, Gemmatimonadota*	[8]
					Bacteroidetes	+ 1.37% distribution		

Soil/ plant class	Biochar category	MPs parent	Efficiency (%)				Note	Reference
			Genes/Enzyme		Bacteria			
			Kind	*Tend	Kind	*Tend		
							are dominant bacterial phyla.<br>• Biochar treatment can enhance disease resistance in pepper plants and promote root growth.	
Paddy rice field	Magnetic biochar - derived rice straw	PE, PVC	Dehalogenase, benzoate metabolism glyceride and glyceophospholipid,	+	*Candidatus Saccharibacteria, Bacteroidetes*	+	• MEBC promotes nutrient transport and the degradation of	[1]

Soil/ plant class	Biochar category	MPs parent	Efficiency (%)				Note	Reference
			Genes/Enzyme		Bacteria			
			Kind	*Tend	Kind	*Tend		
	and ferrous		ABC transporter genes				microplastic products.	
			alkane monooxygenase, PVA dehydrogenase, alkane dehalogenase	–	*Proteobacteria*	–	• MEBC enhances the growth of benzoate-metabolizing bacteria.	
Agricultural sugarcane soil	Corn stalk (600 °C)	HDPE	Diversity index of 16S rRNA gene	OTUs: + (6.28 – 8.00%)<br>Chao1: + (6.42–7.98%)	Diversity index based on phoD-harboring bacteria	OTUs: – (2.3 -3.67%)<br>Chao1: – (1.54-2.59%)	Biochar increases the relative abundance of Subgroup_10, *Bacillus, Pseudomonas, Amycolatopsis and*	[14]

Soil/ plant class	Biochar category	MPs parent	Efficiency (%)				Note	Reference
			Genes/Enzyme		Bacteria			
			Kind	*Tend	Kind	*Tend		
				Shannon: + (3.8 -4.48%)		Shannon: – (1.52 – 1.85)%	*Bradyrhizobium* in soil microbes, inhibiting harmful bacteria and promoting plant growth.	

***Tend refers to the observed trend in microbial abundance and soil biological indicators.*

+ indicates an increase, while – indicates a decrease.

Table S3: Biochar application for MP removal in water and wastewater.

Wastewater/ microplastic	Biochar	Model	Efficiency (%)	Q_e (mg/g)	Kinetic	Mechanism (optimum conditions)	Reference
PS sphere (10 µm)	Corn straw and sand (300-500°C)	Filter column	99	-	-	• Stuck, Trapped, Entangled. Higher pH in effluent: BC-500 > BC-400 > BC-300 > silica sand.	[9]
PS particles (1 µm)	London Plane bark biochar	Batch	-	60.05	Second order: k = 0.023 g/mg/h	• π-π interaction between PS benzene rings and BC (pH 5)	[15]

Wastewater/ microplastic	Biochar	Model	Efficiency (%)	Q_e (mg/g)	Kinetic	Mechanism (optimum conditions)	Reference
					q_e = 59.9 mg/g		
Rigid polystyrene (PS) (75, 150, 300 µm diameter)	Banana peel (650 °C)	Filter column	100	-	-	• Filtration, pore filling, H-bonding, hydrophobic interaction. Efficiency drops after 17h continuous filtration.	[16]
PS and carboxylate functional	Sand + 1% BC from cellulose (Pyrolysis 700°C)	Filter column	99.6	-	-	• Biochar reduces negative charge of MPs in wastewater,	[17]

Wastewater/ microplastic	Biochar	Model	Efficiency (%)	Q_e (mg/g)	Kinetic	Mechanism (optimum conditions)	Reference
group (<10 µm)						• Increasing retention efficiency. Decreased porosity enhances MPs capture.<br>• Complex surface structure improves retention efficiency.	
Polystyrene (10 µm)	Sawdust BC – modified magnatic	Batch	94.81-99.46	374.57	Second order: k = 2.82 g/mg/h	• Enhanced adsorption via electrostatic interactions. Modified	[11]

Wastewater/ microplastic	Biochar	Model	Efficiency (%)	Q_e (mg/g)	Kinetic	Mechanism (optimum conditions)	Reference
					q_e = 100.60 mg/g	biochar shows better adsorption for $H_2PO_4^-$ ions.<br>• Optimal pH 7. Efficiency > 90% even at pH 9.<br>• Electrostatic interaction and metal-O-PS bonds interaction	
	Sawdust BC – modified magnatic and Mg			334.03	Second order: k = 3.72 g/mg/h q_e = 98.52 mg/g		
	Sawdust BC – modified magnatic and Mg			355.72	Second order: k = 3.72 g/mg/h		

Wastewater/ microplastic	Biochar	Model	Efficiency (%)	Q_e (mg/g)	Kinetic	Mechanism (optimum conditions)	Reference
					q_e = 99.21 mg/g		
Polyamide (aged) (27–307 µm)	Magnetic corncob	Batch	97	1145 – 1173	Elovich kinetic: (R^2 = 0.93 – 0.96) α = 61.84 – 256.9 mg/g/min β = 0.06 – 0.0086 g/mg	• Crystallinity reduction, increased oxygen groups. Optimum pH 5-9. • Optimum pH 5-9. Efficiency highest at pH 5. • Electrostatic shielding, "salting-out" effect and	[18]

**Wastewater/ microplastic**	**Biochar**	**Model**	**Efficiency (%)**	**Q_e (mg/g)**	**Kinetic**	**Mechanism (optimum conditions)**	**Reference**
					Second order: (R^2 = 0.73 – 0.88) k = 0.009 – 0.0012 g/mg/h q_e = 151.7 – 991.9 mg/g	buffer effect maintain adsorption efficiency.	
Polystyrene (1 µm)	Magnetic from pine sawdust	Batch	96.2	5.5	Second order:		[19]

Wastewater/ microplastic	Biochar	Model	Efficiency (%)	Q_e (mg/g)	Kinetic	Mechanism (optimum conditions)	Reference
(20 mg/L)					k = 0.152 g/mg/h<br>q_e = 6.388 mg/g	• Adsorption via electrostatic interactions and metal-O-PS-MP formation (pH 3-7).<br>• Adsorption efficiency decreases with anion presence (PO_4^{3-}, CO_3^{2-}, HCO_3^{-}).	
	Magnetic from pine sawdust and Zn	Batch	84.8	-	-		
Monodispersed polystyrene (6 µm)	Amino-functionalized zeolite	Batch	79 - 94	4.54 – 4.63	Second order: (R^2 = 0.99)	• π-π interactions, electrostatic forces,	[20]

Wastewater/ microplastic	Biochar	Model	Efficiency (%)	Q_e (mg/g)	Kinetic	Mechanism (optimum conditions)	Reference
	series/phosphoric acid-coffee waste biochar (AFZ)				k = 0.78 g/mg/h<br>q_e = 4.653 mg/g	hydrogen bonding, Van der Waals forces.<br>• Functional moieties such as C–H, C–O, C=C, N–H, Al–O and Si–O was majorly responsible for the adsorption process.<br>• pH optimize = 7	
					Mixed-1,2-order:<br>(R^2 = 1.0)<br>k = 0.42 g/mg/h<br>q_e = 4.898 mg/g		

Wastewater/ microplastic	Biochar	Model	Efficiency (%)	Q_e (mg/g)	Kinetic	Mechanism (optimum conditions)	Reference
Agriculture runoff (LDPE, PET, PA, PC, PE, PS, PVC) (> 40 µm)	Sugarcane & pinewood	Filter column	86.6 - 92.6	-	-	• Pinewood BC (900°C) shows better trapping and adsorption due to hydroxyl groups. Efficiency inversely proportional to MP size.	[21]
Polyethylene (10 µm, 2 – 3 mm)	Palm kernel shell / Coconut shell	Filter column	92.3%-94.1%	-	-	• Hydrophobic interactions enhance clustering. Smaller MP particles reduce adsorption efficiency.	[10]

Wastewater/ microplastic	Biochar	Model	Efficiency (%)	Q_e (mg/g)	Kinetic	Mechanism (optimum conditions)	Reference
Polystyrene (1 – 1000 nm)	Peanut shells	Upflow filter	16,47%	-	-	• Rough surface and high SSA improve MP retention.<br>• Humic acid reduces adsorption efficiency.<br>• Calcium ions reduce biochar efficiency compared to sodium ions.	[13]
	Magnatic peanut shells		40,96%	-	-		
Polystyrene (100 nm)	Lignin-modified preparation of	Batch	97.87	353.15	Second order:	• Nanoplastic adsorption strongly influenced by acidic conditions due to	[22]

Wastewater/ microplastic	Biochar	Model	Efficiency (%)	Q_e (mg/g)	Kinetic	Mechanism (optimum conditions)	Reference
	spontaneous magnetic sludge				k = 0.0666 – 0.0948 g/mg/min<br>q = 155.55 – 168.93 mg/g<br>(Temp: 288 – 308 K)	significant zeta potential changes.<br>• Optimum adsorbent dosage: 0.6 g/L. Oxygen-rich functional groups facilitate hydrogen bonding with MPs.<br>• Metal oxides in MBC also form metal-O-PS bonds.	

Table S4: Comparison between BC and another method to remove MP from aqueous environments.

Method	Microplastic	Efficiency (%)	Mechanism	Condition	References
Algal (*Chlorella Vulgaris*)	PS (65.49-328.40 µm)	73.01	Bio – coagulation	• pH: 7.5<br>• MP concentration: 789.37 mg/L<br>• Time: 31.9 min<br>• Biomass: 274.05 mg/L	[23]
Algal (*Spirulina platensis*)	PS (328.4 µm)	81	Bio – coagulation	• pH: 4<br>• MP concentration: 500 mg/L<br>• Time: 30 min<br>• Biomass: 250 mg/L	[24]
Activated jute stick charcoal	PVC (250 µm)	94.12	Adsorption	• MPs (5 g/L)<br>• pH 7	[25]

				• Time = 120 min<br>• q_{max} = 4.4668 mg/g<br>• Dose = 10g/L	
Abelmoschus esculentus *(*Okra)	PVC (<85 µm)	80.11	Bio – coagulation	• Dose: 70 mg/L<br>• pH 10<br>• MP concentration: 20 mg/L	[26]
	PS (<100 µm)	64.46		• Dose: 70 mg/L<br>• pH 3<br>• MP concentration: 20 mg/L	
Electro-coagulation (Al electrode)	PE (286.7 µm)	93.2	• Adsorption by low degree of polymerization flocculant	• Electrolyte concentration: 0.05M<br>• pH 7.2<br>• Voltage density = 10V	[27]
	PMMA (6.3 µm)	91.7			
	CA	98.2			

	(1–2 mm)		• MP captured and sweeped by high degree of polymerization flocculant	• Time: 6 h<br>• MP concentration = 0.5 g/L	
	PP (1–2 mm)	98.4			
Electro-coagulation/ Electro-flocculation (Al anode -Fe cathode electrodes)	PE (150 µm) and PVC (250 µm)	100%	MP captured and sweeped by $Al(OH)_3$ and $Fe(OH)_2$	• pH 7<br>• Time: 20 min<br>• Current density = 20 A/m^2	[28]
PVDF membrane Hydrophobic membrane 0.22 µm)		100%	Filter by pore size	• pH 7<br>• Pressure 2 bar	

References

1. Ji, M.; Giangeri, G.; Yu, F.; Sessa, F.; Liu, C.; Sang, W.; Canu, P.; Li, F.; Treu, L.; Campanaro, S. *J. Hazard. Mater.* **2023,** *458,* 131950. doi:10.1016/j.jhazmat.2023.131950
2. Palansooriya, K. N.; Sang, M. K.; Igalavithana, A. D.; Zhang, M.; Hou, D.; Oleszczuk, P.; Sung, J.; Ok, Y. *Environ. Res.* **2022,** *209,* 112807. doi:10.1016/j.envres.2022.112807
3. Yang, L.; Shen, P.; Liang, H.; Wu, Q. *Ecotoxicol. Environ. Saf.* **2024,** *271,* 115935. doi:10.1016/j.ecoenv.2024.115935
4. Su, X.; Qian, F.; Bao, Y. *Environ. Res.* **2024,** *240,* 117488. doi:10.1016/j.envres.2023.117488
5. Palansooriya, K. N.; Withana, P. A.; Jeong, Y.; Sang, M. K.; Cho, Y.; Hwang, G.; Chang, S.; Ok, Y. *Appl. Biol. Chem.* **2024,** *67,* 3. doi:10.1186/s13765-023-00851-w
6. Yu, H.; Pu, Z.; Wang, S.; Chen, Y.; Wang, C.; Wan, Y.; Dong, Y.; Wang, J.; Wan, S.; Wang, D.; Xie, Z. *Sci. Total Environ.* **2024,** *932,* 172927. doi:10.1016/j.scitotenv.2024.172927
7. Khalid, A. R.; Shah, T.; Asad, M.; Ali, A.; Samee, E.; Adnan, F.; Bhatti, M. F.; Marhan, S.; Kammann, C. I.; Haider, G. *Environ. Pollut.* **2023,** *332,* 121810. doi:10.1016/j.envpol.2023.121810
8. Ran, T.; Li, J.; Liao, H.; Zhao, Y.; Yang, G.; Long, N. *Environ. Technol. Innovation* **2023,** *31,* 103174. doi:10.1016/j.eti.2023.103174
9. Wang, Z.; Sedighi, M.; Lea-Langton, A. *Water Res.* **2020,** *184,* 116165. doi:10.1016/j.watres.2020.116165
10. Hanif, M. A.; Ibrahim, N.; Dahalan, F. A.; Md. Ali, U. F.; Hasan, M.; Azhari, A. W.; Jalil, A. *Environ. Sci. Pollut. Res.* **2023,** *30,* 60106–60120. doi:10.1007/s11356-023-26741-8
11. Wang, J.; Sun, C.; Huang, Q.; Chi, Y.; Yan, J. *J. Hazard. Mater.* **2021,** *419,* 126486. doi:10.1016/j.jhazmat.2021.126486

12. Wu, C.; Ma, Y.; Shan, Y.; Song, X.; Wang, D.; Ren, X.; Hu, H.; Cui, J.; Ma, Y. *Chemosphere* **2024,** *362,* 142698. doi:10.1016/j.chemosphere.2024.142698
13. Wang, X.; Dan, Y.; Diao, Y.; Liu, F.; Wang, H.; Sang, W.; Zhang, Y. *Sci. Total Environ.* **2022,** *847,* 157576. doi:10.1016/j.scitotenv.2022.157576
14. Wu, Q.; Zhou, W.; Chen, D.; Tian, J.; Ao, J. *Plants* **2023,** *13,* 83. doi:10.3390/plants13010083
15. Zhang, X.; Lv, D.; Liu, Z.; Xu, D.; Yang, F.; Wang, X.; Tan, Z.; Gao, W.; Liu, R.; Su, C. *Colloids Surf., A* **2024,** *694,* 134159. doi:10.1016/j.colsurfa.2024.134159
16. Subair, A.; Krishnamoorthy Lakshmi, P.; Chellappan, S.; Chinghakham, C. *Environ. Sci. Pollut. Res.* **2024,** *31,* 13753–13765. doi:10.1007/s11356-024-32088-5
17. Hsieh, L.; He, L.; Zhang, M.; Lv, W.; Yang, K.; Tong, M. *Water Res.* **2022,** *221,* 118783. doi:10.1016/j.watres.2022.118783
18. Li, J.; Chen, X.; Yu, S.; Cui, M. *Sci. Total Environ.* **2023,** *875,* 162647. doi:10.1016/j.scitotenv.2023.162647
19. Zhang, L.; Zhang, Q.; Wang, Y.; Cui, X.; Liu, Y.; Ruan, R.; Wu, X.; Cao, L.; Zhao, L.; Zheng, H. *J. Environ. Manage.* **2023,** *347,* 119158. doi:10.1016/j.jenvman.2023.119158
20. Omorogie, M.; Helmreich, B. *Ind. Eng. Chem. Res.* **2024,** *63,* 3947–3961. doi:10.1021/acs.iecr.3c03971
21. Olubusoye, B. S.; Cizdziel, J. V.; Wontor, K.; Heinen, E.; Grandberry, T.; Bennett, E. R.; Moore, M. T. *Front. Environ. Sci.* **2024,** *12,* 1388606. doi:10.3389/fenvs.2024.1388606
22. Feng, D.; Yi, J.; Liu, Y.; Fu, J.; Gong, R.; Liu, P.; Guo, J.; Cui, K.; Li, H. *J. Chem. Technol. Biotechnol.* **2025,** *100,* 778–791. doi:10.1002/jctb.7819
23. Lotfigolsefidi, F.; Davoudi, M.; Sarkhosh, M.; Bonyadi, Z. *Sci. Rep.* **2025,** *15,* 501. doi:10.1038/s41598-024-84114-8
24. Eydi Gabrabad, M.; Yari, M.; Bonyadi, Z. *Sci. Rep.* **2024,** *14,* 2506. doi:10.1038/s41598-024-53123-y

25. Alom, N.; Roy, T.; Sarkar, T.; Rasel, M.; Hossain, M. S.; Jamal, M. *Heliyon* **2024,** *10,* e37380. doi:10.1016/j.heliyon.2024.e37380
26. Eydi Gabrabad, M.; Bonyadi, Z.; Davoudi, M.; Barikbin, B. *Appl. Water Sci.* **2024,** *14,* 217. doi:10.1007/s13201-024-02249-5
27. Shen, M.; Zhang, Y.; Almatrafi, E.; Hu, T.; Zhou, C.; Song, B.; Zeng, Z.; Zeng, G. *Chem. Eng. J.* **2022,** *428,* 131161. doi:10.1016/j.cej.2021.131161
28. Akarsu, C.; Kumbur, H.; Kideys, A. *Water Sci. Technol.* **2021,** *84,* 1648–1662. doi:10.2166/wst.2021.356
